# Degenerative lamellar macular holes: tractional development and morphological alterations

**DOI:** 10.1007/s10792-020-01674-0

**Published:** 2021-01-12

**Authors:** Andreas Bringmann, Jan Darius Unterlauft, Renate Wiedemann, Thomas Barth, Matus Rehak, Peter Wiedemann

**Affiliations:** Department of Ophthalmology and Eye Hospital, University of Leipzig, Liebigstraße 12, 04103 Leipzig, Germany

**Keywords:** Fovea, Lamellar macular hole, Full-thickness macular hole, Müller glia, Müller cell cone

## Abstract

**Purpose:**

The development of degenerative lamellar macular holes (DLH) is largely unclear. This study was aimed at documenting with spectral-domain optical coherence tomography the tractional development and morphological alterations of DLH.

**Methods:**

A retrospective case series of 44 eyes of 44 patients is described.

**Results:**

The development of DLH is preceded for months or years by tractional deformations of the fovea due to the action of contractile epiretinal membranes (ERM) and/or the partially detached posterior hyaloid, or by cystoid macular edema (CME). DLH may develop after a tractional stretching and thickening of the foveal center, from a foveal pseudocyst, after a detachment of the foveola from the retinal pigment epithelium, a disruption of the foveal structure due to CME, and after surgical treatment of tractional lamellar or full-thickness macular holes (FTMH). The foveal configuration of a DLH can be spontaneously reestablished after short transient episodes of CME and a small FTMH. A DLH can evolve to a FTMH by traction of an ERM. Surgical treatment of a DLH may result in an irregular regeneration of the foveal center without photoreceptors.

**Conclusions:**

Tractional forces play an important role in the development of DLH and in the further evolution to FTMH. It is suggested that a DLH is the result of a retinal wound repair process after a tractional disruption of the Müller cell cone and a degeneration of Henle fibers, to prevent a further increase in the degenerative cavitations.

## Introduction

Various macular diseases are associated with anteroposterior or tangential traction exerted by the partially detached posterior hyaloid and/or contractile epiretinal membranes (ERM). Tractional forces may cause a disruption of the foveal integrity resulting in the formation of a partial- or full-thickness macular defect. Full-thickness macular holes (FTMH) develop by a disruption of both the Müller cell cone [1–4] and the external limiting membrane (ELM) in the foveola. The common feature of most types of partial-thickness macular defects is a disruption of the Müller cell cone or of the connection between the cone and the foveal walls which results in a deformation of the foveal pit; the (outer part of the) central outer nuclear layer (ONL) and the ELM are not disrupted and keep the foveal walls together preventing the formation of a FTMH [5]. In outer lamellar holes, a special type of partial-thickness macular defects, the outer layers of the foveola including the ELM are disrupted and the nondisrupted horizontal layer of the Müller cell cone keeps the foveal walls together [6].

Partial-thickness macular defects are grossly classified into macular pseudoholes (MPH), foveal pseudocysts, tractional lamellar holes (TLH), degenerative lamellar holes (DLH), and outer lamellar holes; mixed types of TLH and DLH were also described [5–10]. TLH are characterized by a disruption of the Müller cell cone in the foveola and an elevation of the inner layers of the foveal walls which results in an intraretinal splitting (schisis) between the outer plexiform layer (OPL) and Henle fiber layer (HFL) of the foveal walls and parafovea [5, 9, 10]. The main characteristic of DLH is the development of degenerative cavitations into the lower foveal walls [8–12]; this proceeds by a slow and chronic degeneration of the HFL, OPL, and inner nuclear layer (INL) of the foveal walls and parafovea and is associated with a degeneration of the ONL and photoreceptor layer in the foveola [5, 9, 13]. The spaces left by the degenerated photoreceptor cells in the foveola are filled by proliferating cells of the disrupted Müller cell cone [5]. DLH often shows the development of a nonproliferative, nontractional, yellowish epiretinal tissue, termed lamellar hole-associated epiretinal proliferation (LHEP), on top of the nerve fiber layer (NFL) of the foveal walls and parafovea [9–11, 14–20]. In spectral-domain optical coherence tomography (SD-OCT) images, LHEP is composed of a tissue of medium reflectivity and hyperreflective layers at the vitreal and retinal sides of this tissue. Connections between Müller cells in the foveola and LHEP suggest that, in addition to vitreal cells like fibroblasts and hyalocytes, cells of the disrupted Müller cell cone contribute to the development of LHEP [5, 15, 17, 18, 21]. Because tractional ERM and LHEP often coexist in DLH, ERM may contribute to the development of LHEP [5, 13].

TLH arise by tractional forces of ERM and/or the partially detached posterior hyaloid [13, 15, 19]. The traction causes a disruption of the Müller cell cone in the foveola, an elevation of the inner layers of the foveal walls, and a schistic tissue splitting between the OPL and HFL [5]. The pathogenesis of DLH is unclear. DLH was suggested to be formed by a slow and chronic degenerative process [9]. Another study proposed that DLH is generated by various pathogenic processes: an initial tractional disruption of the Müller cell cone or of the connection between the Müller cell cone and the foveal walls produces a small schisis between the OPL and HFL; this is followed by a degenerative enlargement of the schisis resulting in the formation of the cavitations in the lower foveal walls which is associated with the degeneration of the central photoreceptors and the development of LHEP [5]. In the present study, we document different modes of a tractional development of DLH by SD-OCT. Usually, DLH are considered to be morphologically stable and display only slow structural modifications over long time periods [8, 12, 17, 22–25]. We describe two cases of relatively fast transient morphological alterations of DLH which include the transient disruption of the foveal structure due to cystoid macular edema (CME) and the formation of a small FTMH. In addition, we describe DLH formation after closure of a FTMH, the evolution of a DLH into a FTMH, and the foveal regeneration after surgical removal of LHEP.

## Methods

This is a retrospective, single-center chart review. The study followed the ethical standards of the 1964 Declaration of Helsinki and its later amendments. The protocol was approved by the Ethics Committee of the Medical Faculty of the University of Leipzig (#143/20-ek). The ethics committee is registered as Institutional Review Board at the Office for Human Research Protections (registration number, IORG0001320/IRB00001750). We retrospectively reviewed charts of patients who were referred to the Department of Ophthalmology, University of Leipzig, Germany, between August 2008 and January 2020. Forty-four patients with a DLH and other types of macular defects, which developed into a DLH during the examination period, were included in the study. Cross-sectional images of the macula were obtained with SD-OCT (Spectralis, Heidelberg Engineering, Heidelberg, Germany). Best-corrected visual acuity (BCVA) was determined with a Snellen chart and is given in decimal units.

**Fig. 1:**
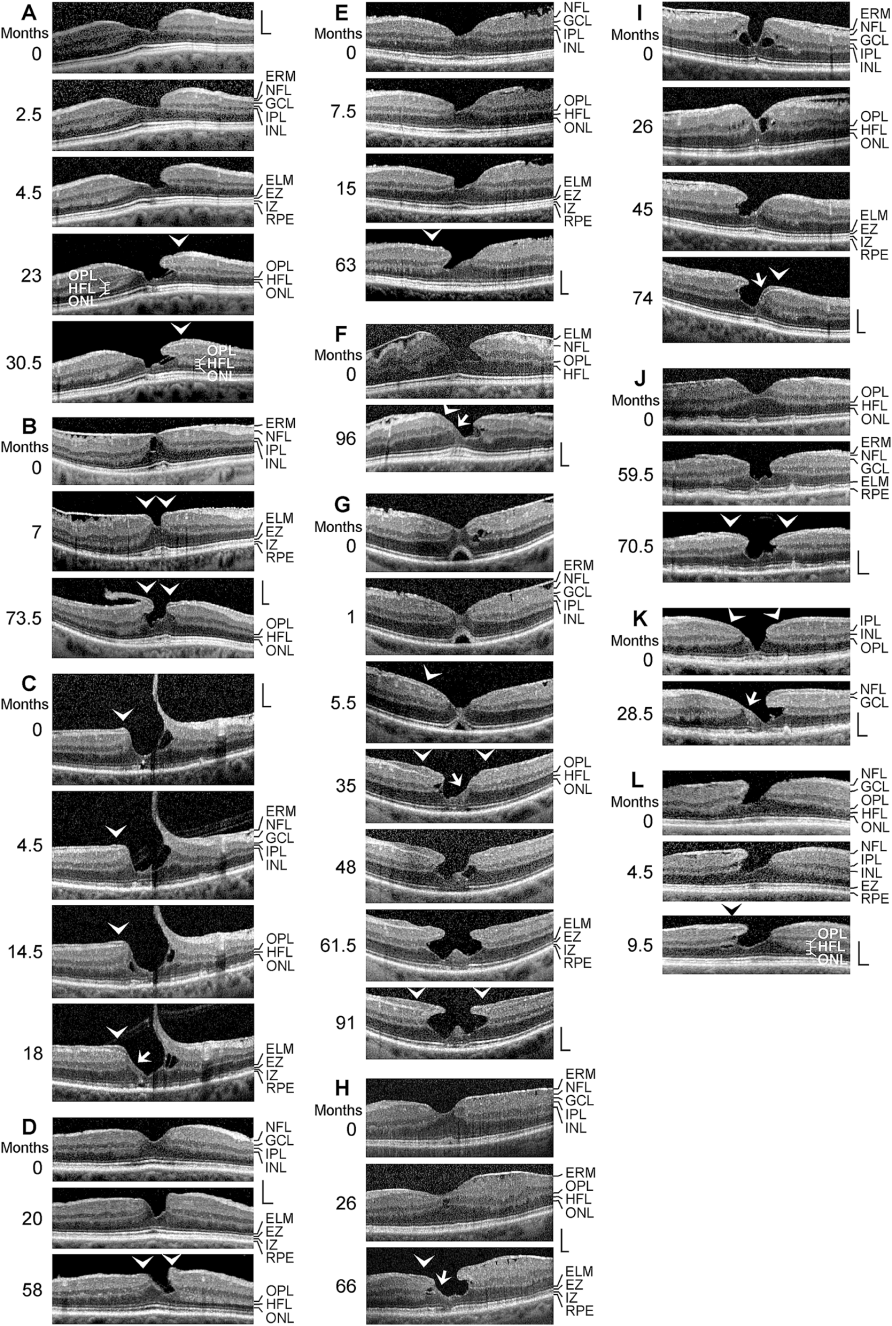
Tractional development of degenerative lamellar holes (DLH). The images show SD-OCT scans through the fovea and parafovea of 12 eyes of 12 patients. The months after the first visit (0) are indicated *left* of the images. The *arrowheads* indicate lamellar macular hole-associated epiretinal proliferation (LHEP). The *arrows* indicate morphological connections between Müller cells in the foveola and LHEP. **i** Development of a DLH after surgical treatment of a tractional lamellar hole in an eye with macular pucker. Vitrectomy with internal limiting membrane and epiretinal membrane (ERM) peeling was performed 2.5 months after the first visit. Scale bars, 200 µm. ELM, external limiting membrane; ERM, epiretinal membrane; EZ, ellipsoid zone; GCL, ganglion cell layer; INL, inner nuclear layer; HFL, Henle fiber layer; IPL, inner plexiform layer; IZ, interdigitation zone; NFL, nerve fiber layer; ONL, outer nuclear layer; OPL, outer plexiform layer; RPE, retinal pigment epithelium

**Fig. 2:**
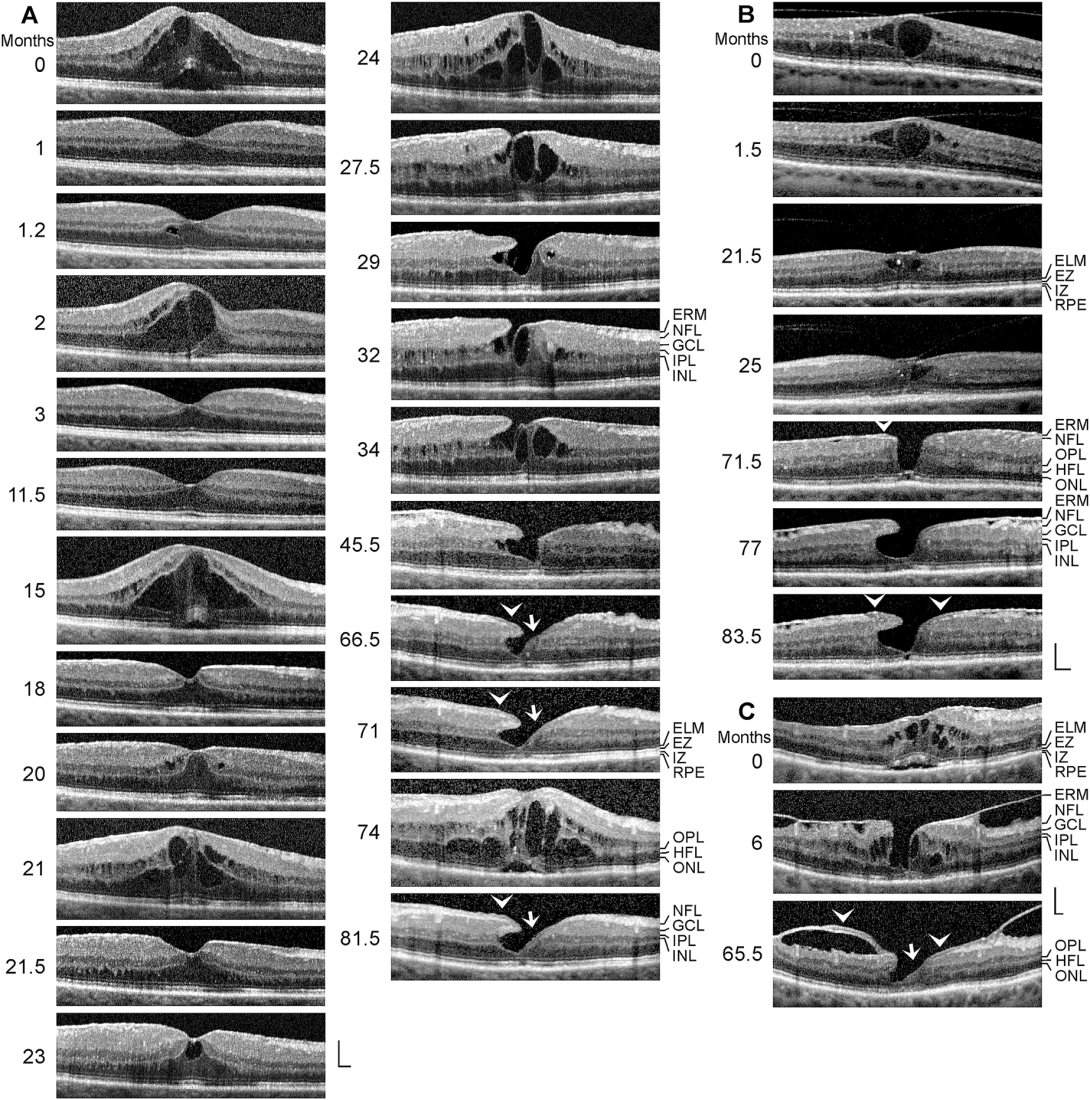
Cystoid macular edema may precede the development of a degenerative lamellar macular hole. **a**–**c** The images show SD-OCT scans through the fovea and parafovea of 3 eyes of 3 patients. The months after the first visit (0) are indicated *left* of the images. The *arrowheads* indicate lamellar macular hole-associated epiretinal proliferation. The *arrows* indicate morphological connections between Müller cells in the foveola and the edges of nonelevated foveal walls. Scale bars, 200 µm. ELM, external limiting membrane; ERM, epiretinal membrane; EZ, ellipsoid zone; GCL, ganglion cell layer; HFL, Henle fiber layer; INL, inner nuclear layer; IPL, inner plexiform layer; IZ, interdigitation zone; NFL, nerve fiber layer; ONL, outer nuclear layer; OPL, outer plexiform layer; RPE, retinal pigment epithelium

**Fig. 3:**
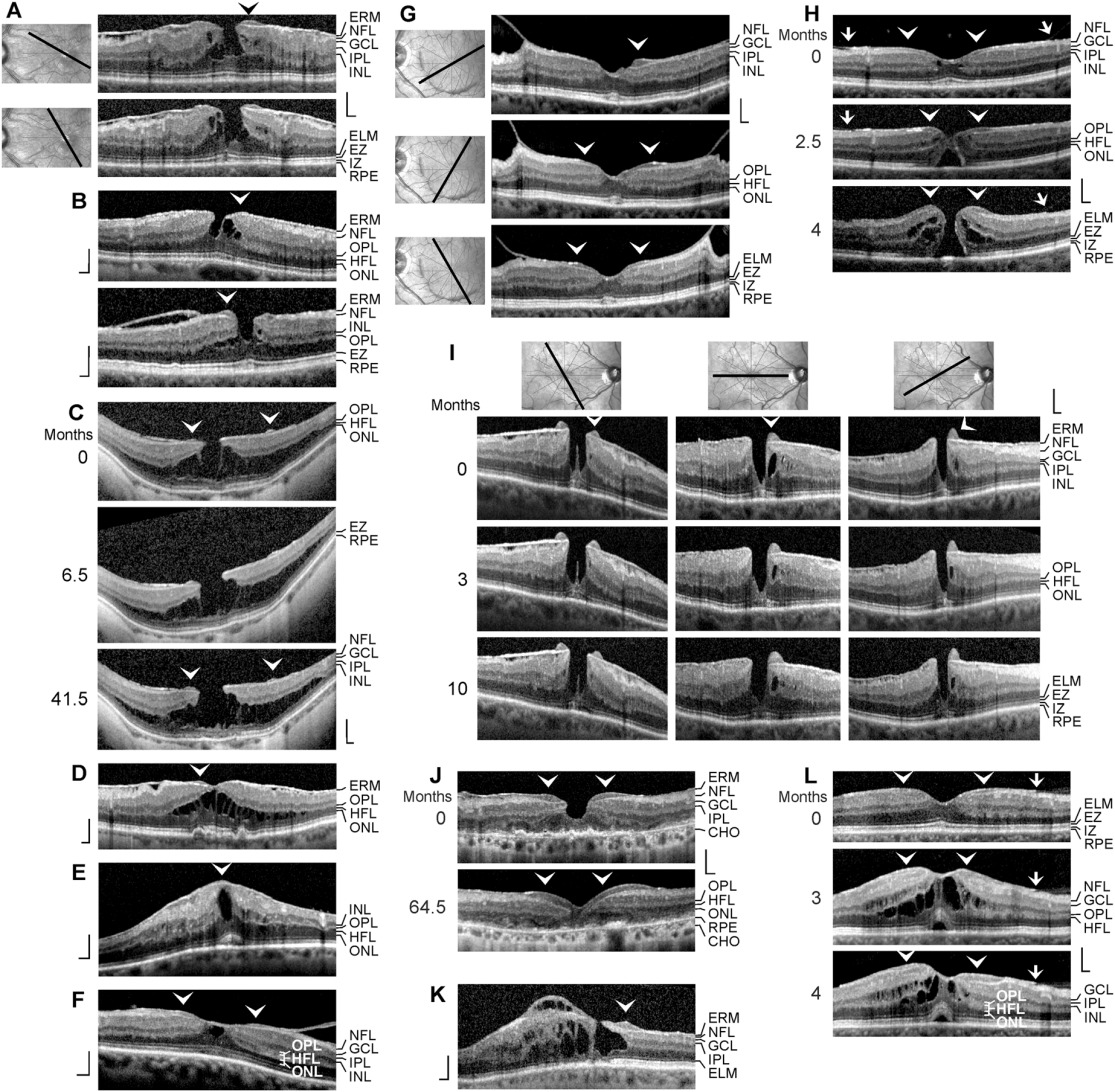
Lamellar macular hole-associated epiretinal proliferation (LHEP) in various types of foveal defects. The images show SD-OCT scans through the fovea and parafovea of 13 eyes of 13 patients. The months after the first visit (0) are indicated *left* of the images. The orientations of the scans are shown *above* or *left* of the images. The *arrowheads* indicate LHEP. **a, b** Macular pseudoholes (MPH) with cleaved edges. **c** A tractional lamellar hole (TLH) with LHEP in an eye with high myopia. **d**–**f** Foveal pseudocysts. **g** Vitreomacular traction. **h** Development of a full-thickness macular hole by tangential traction exerted by the partially detached posterior hyaloid which adhered at the perifovea (*arrows*). The hole formation is associated with the formation of edematous cysts in the foveal walls. **i, j** Mixed types of a MPH and degenerative lamellar hole (DLH). **k** A DLH with cystoid macular edema (CME) in one foveal wall. Note that the ganglion cell layer (GCL) of this wall also contains edematous cysts. **l** Development of a CME from a fovea with LHEP. The *arrow* indicates a connection between the partially detached posterior hyaloid and an epiretinal membrane (ERM). Scale bars, 200 µm. CHO, choroidea; ELM, external limiting membrane; EZ, ellipsoid zone; HFL, Henle fiber layer; INL, inner nuclear layer; IPL, inner plexiform layer; IZ, interdigitation zone; NFL, nerve fiber layer; ONL, outer nuclear layer; OPL, outer plexiform layer; RPE, retinal pigment epithelium

**Fig. 4:**
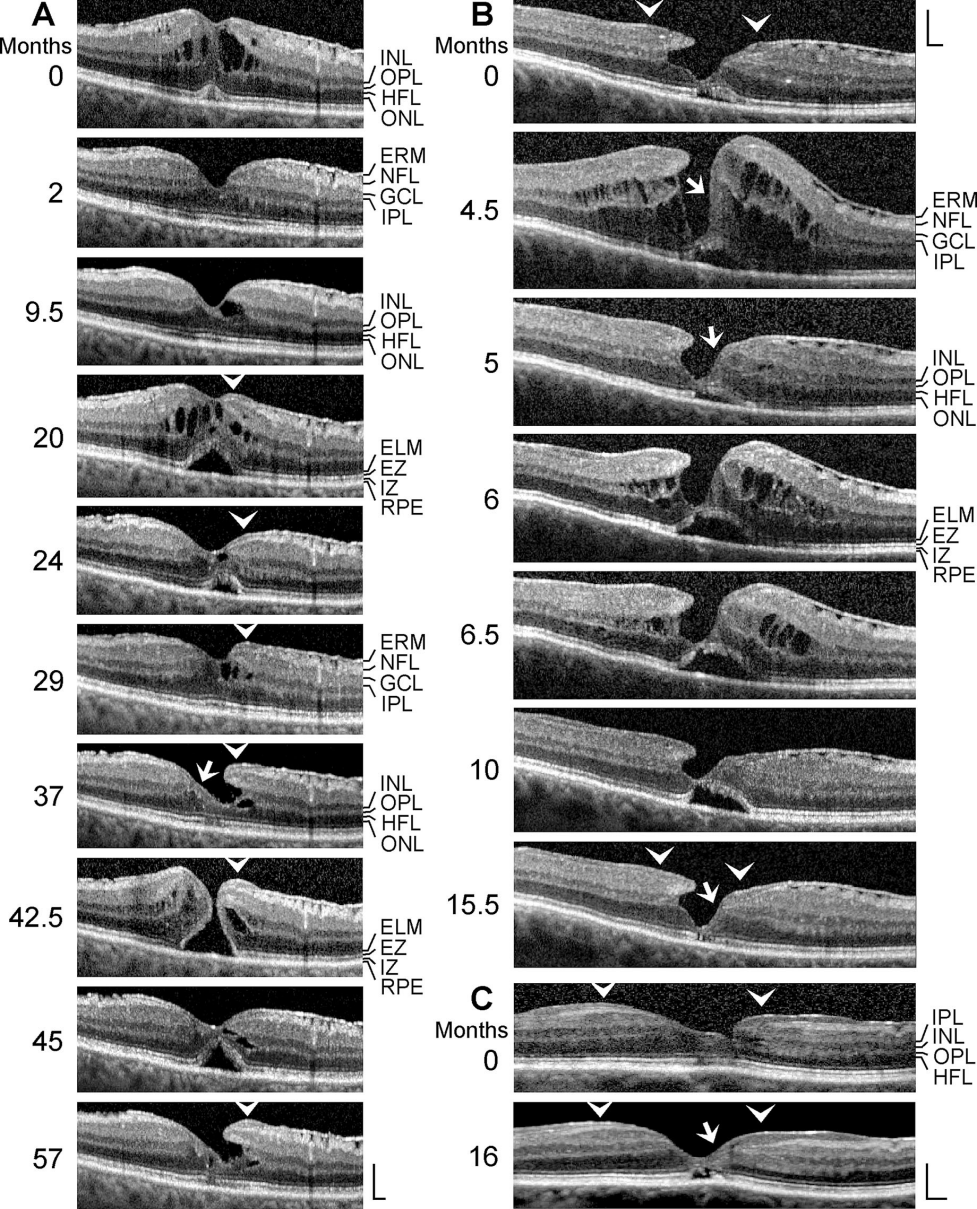
Morphological alterations of degenerative lamellar holes. **a–c** The images show SD-OCT scans through the fovea and parafovea of 3 eyes of 3 patients. The months after the first visit (0) are indicated *left* of the images. The *arrowheads* indicate lamellar macular hole-associated epiretinal proliferation (LHEP). The *arrows* indicate morphological connections between Müller cells in the foveola and LHEP. Scale bars, 200 µm. ELM, external limiting membrane; ERM, epiretinal membrane; EZ, ellipsoid zone; GCL, ganglion cell layer; HFL, Henle fiber layer; INL, inner nuclear layer; IPL, inner plexiform layer; IZ, interdigitation zone; NFL, nerve fiber layer; ONL, outer nuclear layer; OPL, outer plexiform layer; RPE, retinal pigment epithelium

All patients were Caucasians. The time-dependent development of a DLH was observed in 12 eyes of 12 patients (8 women, 4 men; mean ± S.D. age, 68.4 ± 10.8 years; range 41–79 years; Fig. 1a–l). The mean BCVA at the first visit was 0.64 ± 0.20 (range, 0.30–1.00). SD-OCT scans of the macular region of 3 eyes of 3 further patients (1 women, 2 men; mean age, 64.3 ± 11.1 years; range 54–76 years) showed a CME (Fig. 2a–c; mean BCVA at the first visit, 0.41 ± 0.28, range, 0.10–0.63). Thirteen patients with LHEP and various types of foveal defects in one eye were investigated (5 women, 8 men; mean age, 71.0 ± 11.0 years, range 39–79 years; Fig. 3a–l); the BCVA ranged from 0.05 to 0.80 (mean, 0.54 ± 0.25). Morphological alterations of DLH were investigated in 3 eyes of 3 patients (2 woman, 1 man; mean BCVA at the first visit, 0.59 ± 0.07, range, 0.50–0.63; Fig. 4a–c).

**Fig. 5:**
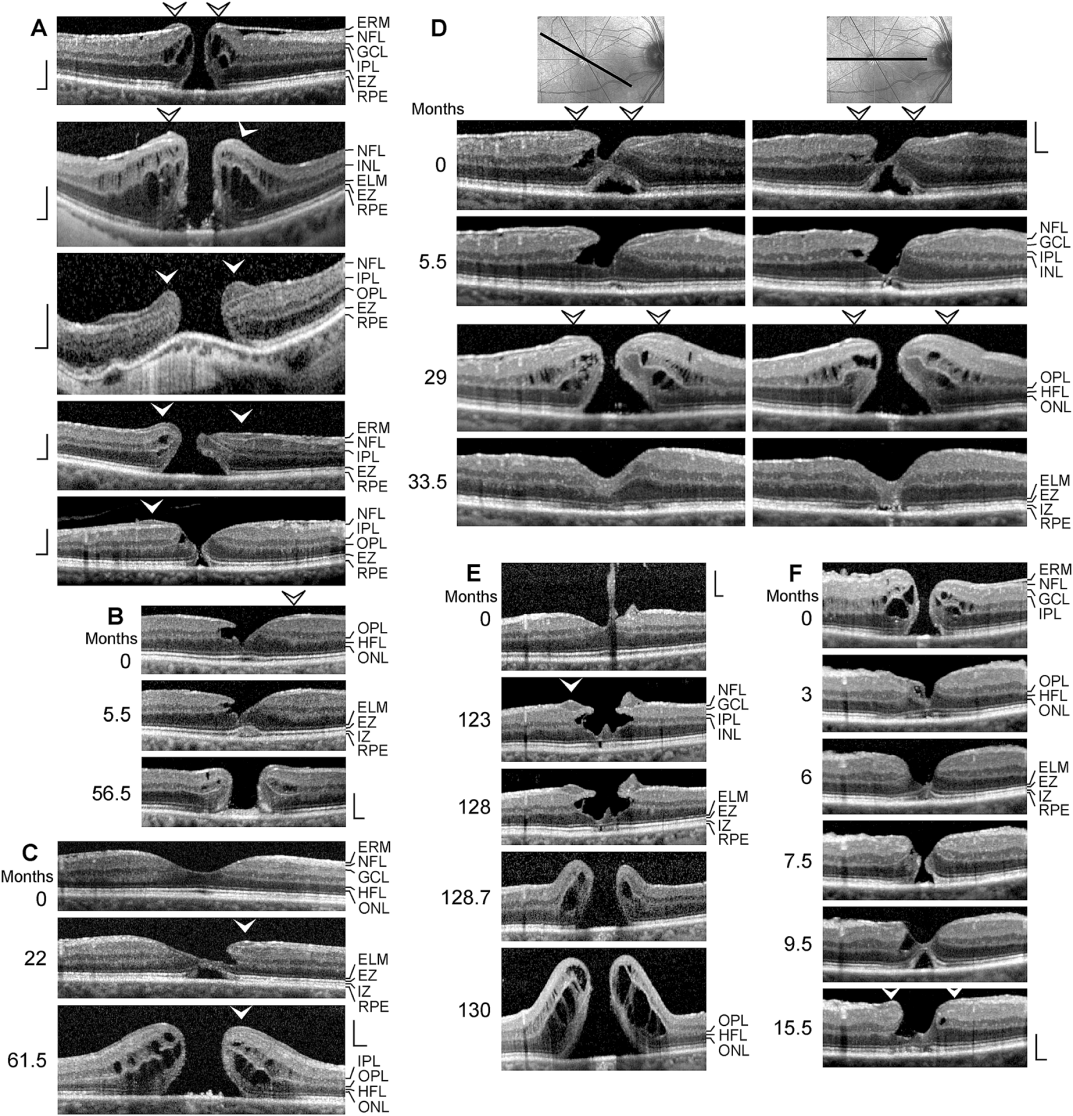
Degenerative lamellar holes (DLH) and full-thickness macular holes (FTMH). The images show SD-OCT scans through the fovea and parafovea of 10 eyes of 10 patients. The months after the first visit (0) are indicated *left* of the images. In **c**, the orientations of the scans are indicated *above* the images. The *arrowheads* indicate lamellar macular hole-associated epiretinal proliferation (LHEP). The *arrows* indicate morphological connections between Müller cells in the foveola and LHEP. **a** Five eyes with a FTMH with LHEP. **b–d** Development of a DLH into a FTMH. **e** Tractional development of a DLH and development into a FTMH. Vitrectomy with ILM and ERM peeling was performed 31 (**d**) and 128.2 months (**e**) after the first visit, respectively. **f** Development of a DLH after surgical closure of a FTMH. Vitrectomy with ILM and ERM peeling was performed 1.5 months after the first visit. Scale bars, 200 µm. ELM, external limiting membrane; EZ, ellipsoid zone; GCL, ganglion cell layer; HFL, Henle fiber layer; INL, inner nuclear layer; IPL, inner plexiform layer; IZ, interdigitation zone; NFL, nerve fiber layer; ONL, outer nuclear layer; OPL, outer plexiform layer; RPE, retinal pigment epithelium

**Fig. 6:**
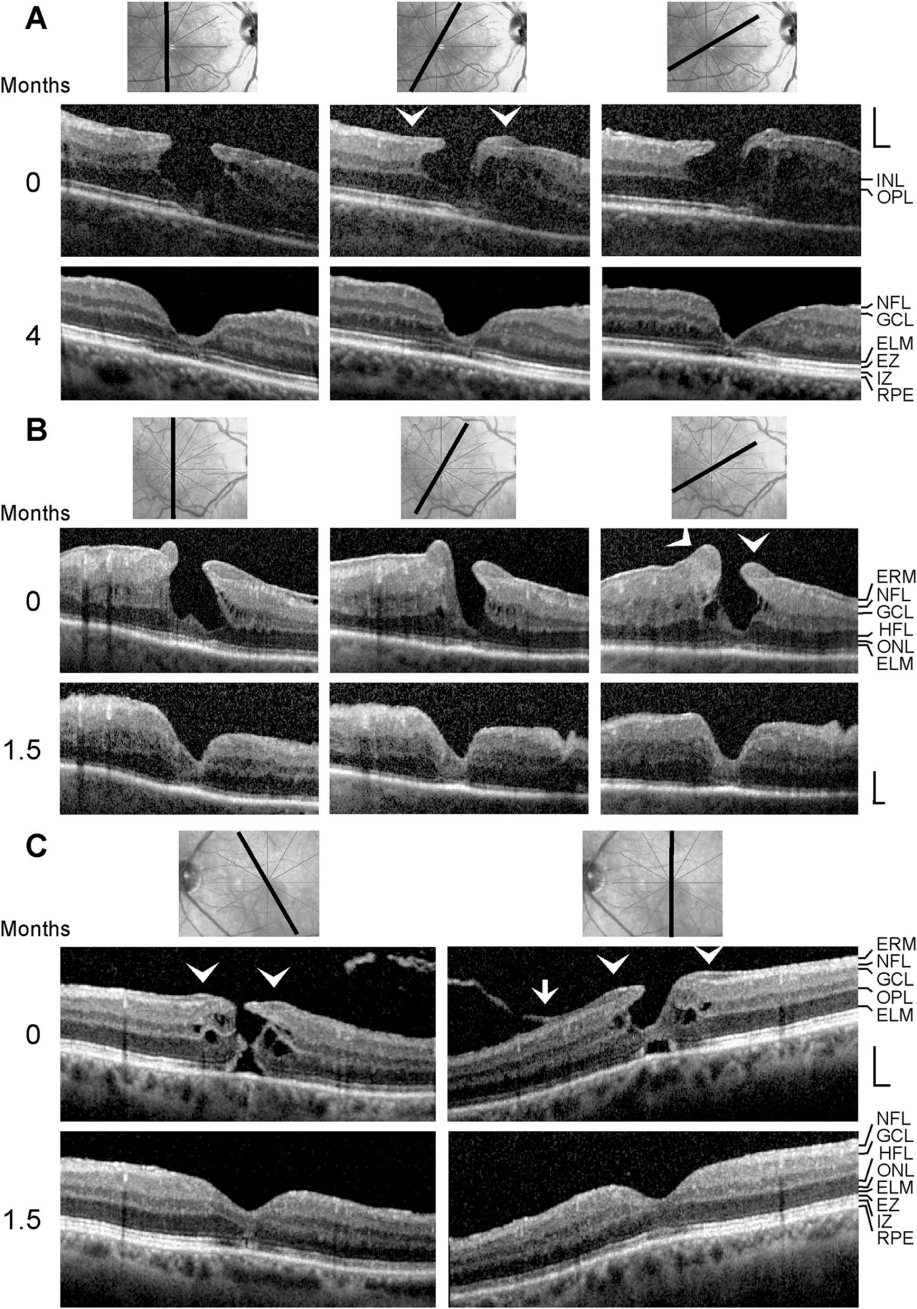
Foveal regeneration after surgical removal of lamellar macular hole-associated epiretinal proliferation (LHEP) by vitrectomy with internal limiting and epiretinal membrane (ERM) peeling. **a–c.** The images show SD-OCT scans through the fovea and parafovea of 3 eyes of 3 patients recorded before (*above*) and after surgery (*below*). The orientations of the scans are shown *above* the images. The months after the first visit (0) are indicated *left* of the images. The *arrowheads* indicate LHEP. The *arrow* indicates the adhesion of the partially detached posterior hyaloid to the parafoveal tissue. Surgery was performed 1.5 months (**a**), one month (**b**), and one day (**c**) after the first visit, respectively. Scale bars, 200 µm. ELM, external limiting membrane; EZ, ellipsoid zone; GCL, ganglion cell layer; HFL, Henle fiber layer; INL, inner nuclear layer; IZ, interdigitation zone; NFL, nerve fiber layer; ONL, outer nuclear layer; OPL, outer plexiform layer; RPE, retinal pigment epithelium

FTMH with LHEP was observed 5 eyes of 5 patients (2 women, 3 men; mean age, 65.6 ± 11.4 years; range 46–74 years; mean BCVA, 0.36 ± 0.36, range, 0.10–1.00; Fig. 5a). The development of a DLH into a FTMH was found in 4 eyes of 4 patients (1 women, 3 men; mean age, 75.5 ± 6.6 years, range 70–85 years; mean BCVA, 0.71 ± 0.11, range, 0.60–0.80; Fig. 5b–e). In two of these cases (Fig. 5d, e), standard pars plana vitrectomy with internal limiting membrane (ILM) and ERM peeling, followed by SF6 tamponade, was carried out 31 and 128.2 months after the first visit, respectively. The development of a DLH after a surgical closure of a FTMH performed 1.5 months after the first visit was investigated in one eye of a 52 year-old man (Fig. 5f); the BCVA improved from 0.30 (first visit) to 0.63 (end of the examination period). The foveal regeneration after surgical removal of LHEP was investigated in 3 eyes of 3 patients (1 women, 2 men; mean ± S.D. age, 62.0 ± 14.1 years; range 47–75 years; Fig. 6a–c). The mean BCVA of these eyes improved slightly from 0.30 ± 0.14 (range, 0.20–0.40) to 0.41 ± 0.30 (range, 0.20–0.63).

## Results

*Tractional development of DLH* The SD-OCT scans shown in Fig. 1 display different cases of a tractional development of DLH. In the fovea shown in Fig. 1a, degenerative cavitations developed in one foveal wall. Tractional forces of an ERM on top of the NFL of this wall caused a deformation of the foveal pit, likely due to a disruption of the connection between the inner Müller cell layer of the foveola to this wall; this resulted in an indentation between the OPL and HFL. Between 2.5 and 4.5 months after the first visit, the indentation developed to a schisis; this was associated with a thinning of the HFL/ONL and a loss of the integrity of the central photoreceptors, as indicated by the hyporeflectivities of the ellipsoid zone (EZ) and interdigitation zone (IZ) lines. Between 4.5 and 23 months, the schisis evolved into a degenerative cavitation which was traversed by thin bundles of Henle fibers. LHEP was formed at the vitreal surface of this foveal wall and parafovea. The central foveola consisted of a tissue of medium reflectivity, likely proliferating cells of the Müller cell cone which fill the photoreceptor cell-free gaps in the foveola. Below this Müller cell tissue, the ELM and EZ lines displayed outward instead of inward deflections (as in the normal fovea). The opposite foveal wall showed no abnormalities with the exceptions of an IZ line defect and the increased reflectivity of the HFL which was likely caused by a tractional distortion of the tissue.

Figure 1b shows the evolution of a foveal pseudocyst (first visit) into a DLH. Tangential traction exerted by ERM caused an anterior stretching of the fovea which produced a thickening of the foveola associated with cyst formation, an elevation of the foveal walls, and a deformation of the fovea externa which is the cone-like arrangement of the elongated photoreceptor segments in the foveola [4]. In addition, there was a loss of the integrity of the outer photoreceptor segments in the left para- and perifovea, as indicated by the absence of the IZ line. Within 7 months after the first visit, the horizontal layer of the Müller cell cone disrupted which caused an indentation at the level of the OPL-HFL interface in the right foveal wall; this was associated with the development of small LHEP at the edges of the foveal walls. Thereafter, a degenerative cavitation developed between the inner plexiform layer (IPL) and ONL of the foveal walls, and the LHEP increased. The ERM continued to the hyperreflective layers of the LHEP.

Figure 1c shows the development of a DLH due to anteroposterior traction which caused a disruption of the inner Müller cell layer of the foveola and an elevation of the inner layers of the foveal walls. LHEP was visible at the edge of a foveal wall. The foveola contained a small photoreceptor- and ONL-free area; this area was filled by a tissue of medium reflectivity. The medium reflectivity and the location suggest that this tissue was formed by Müller cells. The morphology of the lamellar hole did not alter within the next 18 months with the exceptions that the tissue of medium reflectivity, which filled the ONL-free part of the foveola, and the LHEP increased time-dependently. After 18 months, there was a relatively thick connection between the Müller cells in the foveola and the LHEP (*arrow* in Fig. 1c). This was associated with a disappearance of the cyst between the OPL and HFL in this wall.

Figure 1d shows the development of a DLH after a tractional thickening of the central fovea. Within 20 months after the first visit, the contour of the foveal pit was disturbed, likely by a damage to the Müller cell cone. Later on, a schistic cavitation developed between the OPL and HFL of one foveal wall which was associated with the formation of a LHEP at the edges of the walls. Figure 1e shows another case of a tractional deformation of the foveal contour with an indentation between the OPL and HFL of the left foveal wall which developed to a schisis; this was associated with the formation of a small LHEP at the edge of this wall. The fovea shown in Fig. 1f had a schisis between the OPL and HFL in one foveal wall, likely caused by the disruption of the Müller cell cone due to traction of ERM. Within the next 96 months, the schisis developed to a degenerative cavitation; there was an ONL-free area in the central fovea filled by a tissue of medium reflectivity which was connected to the LHEP at the edge of the opposite wall (*arrow* in Fig. 1f).

Figure 1g shows the development of a DLH after a detachment of the foveola from the retinal pigment epithelium (RPE) due to tangential traction exerted by ERM. The detachment of the foveola was associated with a gap in the central ONL which was filled by a tissue of medium reflectivity, likely composed of Müller cells. Between 1 and 5.5 months after the first visit, LHEP developed at the vitreal surface of one foveal wall. The reattachment of the foveola to the RPE between 5.5 and 35 months was associated with the formation of a DLH characterized by the development of a degenerative cavitation, an increase in the LHEP, and a nearly complete absence of photoreceptors in the foveal center which was filled by Müller cells; the Müller cells also covered the central surface of one foveal wall (*arrow* in Fig. 1g). Later on, the degenerative cavitations under the elevated inner layers of the foveal walls time-dependently increased; this was associated with a disruption of the connection between the foveolar Müller cells and the edge of the foveal wall.

Figure 1h shows another case of a DLH formed by tangential traction from an ERM in the parafovea. Contraction of the ERM caused tissue folds at the inner retinal surface and an anterior stretching and thickening of the foveola. A large degenerative cavitation developed via a cystic disruption of the foveola; this was associated with a loss of the integrity of the centralmost photoreceptors (as indicated by the defects of the ELM, EZ, and IZ lines) and the formation of LHEP at the inner surface of one foveal wall. A tissue of medium reflectivity, likely composed of Müller cells, covered the vitreal side of the foveola.

Figure 1i shows the development of a DLH after a surgical treatment of a TLH due to macular pucker. The TLH was characterized by an elevation of the inner layers of the foveal walls (NFL to OPL), a schistic tissue splitting between the OPL and HFL, and cystic cavities in the INL. Vitrectomy with ERM and ILM peeling was performed 2.5 months after the first visit. After surgery, the ERM regrew. Until 45 months, a degenerative cavitation developed in one foveal wall, apparently by the degeneration of the INL and OPL. The opposite foveal wall, which had a LHEP at the inner surface, did not contain degenerative cavitations. An ONL-free part of the foveola was filled by a tissue of medium reflectivity, likely composed of Müller cells; this tissue was connected to the LHEP. Figure 1j shows another eye with a development of DLH after a tractional thickening of the foveal center, apparently due to traction exerted by ERM. The development of degenerative cavitations into the foveal walls was associated with the development of LHEP at the vitreal surface of the walls.

The fovea shown in Fig. 1k was characterized by the absence of the ONL in the foveola and small LHEP at the vitreal surfaces of the foveal walls. The ONL-free area of the foveola was partly filled by a tissue of medium reflectivity; the foveal walls kept together at the ELM. In the further course, large cavitations developed in one foveal wall by the degeneration of the INL and OPL; this was associated with a degeneration of the ONL and enlargements of the LHEP and the tissue of medium reflectivity in the foveola. Figure 1l shows the time-dependent increase in degenerative cavitations in an eye with DLH. The cavitations developed in the foveal wall with the ERM, but not in the opposite wall.

*Development of DLH preceded by CME:* As shown in Fig. 2a–c, the formation of a DLH can be preceded by a CME. In the case shown in Fig. 2a, the first SD-OCT image was recorded 0.5 months after descemet membrane endothelial keratoplasty. In addition, congenital glaucoma was present. Five episodes of CME occurred before the development of a DLH 29 months after the first visit. The CME was characterized by the development of cystic cavities between the OPL and HFL, and within the INL, and large elevations of the inner Müller cell layer of the foveola and the inner layers of the foveal walls (NFL to OPL). These elevations caused a tractional detachment of the central outer retina from the RPE during the first three CME episodes. After the first and second episodes (1 and 3 months), the foveal structure fully recovered. Along with the development of an ERM at the vitreal surface of the parafovea (18 months), the resolution of the edematous cysts did not result in a regular regeneration of the foveal shape. LHEP developed at the vitreal surface of one foveal wall after 24 months. A degenerative cavitation developed after 27.5 months. This was associated with a thinning of the central foveola; after 34 months, the central foveola was composed of the ELM and a thin tissue of medium reflectivity, likely Müller cells. A morphological connection developed between the Müller cells in the foveola and the edge of the nonelevated foveal wall which did not contain a degenerative cavitation (*arrows* in Fig. 2a). After the CME episode at 74 months, the foveal configuration of a DLH was reestablished.

The CME shown in Fig. 2b was induced by traction exerted by the partially detached posterior hyaloid which adhered at the foveola. There was a large cyst in the foveola and a schistic splitting of the foveal walls in the INL. The outer retina in the foveola was only stabilized by the ELM. After resolution of the large central cyst (21.5 months), the inner layer of the foveola still was under tension and hyperreflective. Thereafter, ERM developed at the vitreal surfaces of the foveal walls and parafovea. Contraction of the ERM produced a straightening and thickening of the foveal walls, resembling a MPH [26], which was associated with a centrifugal displacement of the central ONL; the foveal center was composed only by the hyperreflective ELM (71.5 months). Small LHEP developed at the vitreal surfaces of the foveal walls. Thereafter, a large degenerative cavitation developed in one foveal wall due to degeneration of the INL, OPL, and HFL. The outer layer of the elevated foveal wall, which protruded centripetally above the degenerative cavitation, was the IPL.

The CME shown in Fig. 2c was induced by traction exerted by ERM. Between the first visit and 6 months later, the Müller cell cone in the foveola disrupted which was associated with a degeneration of the central outer retina. Thereafter, a degenerative cavitation and LHEP developed. There were continuities between the ERM and the hyperreflective layers of LHEP. The foveal center was composed of a tissue of medium reflectivity, likely Müller cells, which was connected to the LHEP at one foveal wall.

*LHEP in other types of foveal defects* LHEP is most frequently observed in eyes with DLH; some cases of other types of partial-thickness macular defects and FTMH also exhibit LHEP [9–14, 16, 17, 22, 23]. We found LHEP in various types of foveal defects. Figure 3a and b shows MPH with cleaved edges [26], i.e., MPH with a schistic splitting of the foveal walls between the OPL and HFL, but without degenerative cavitations. The holes were induced by ERM traction; the ERM continued into the outer hyperreflective layer of LHEP. A schisis between OPL and HFL, which is produced by an elevation of the inner layers (NFL to OPL) of the foveal walls and parafovea, is also a characteristic of other tractional foveal defects like TLH, whereas a thickening of the foveal walls is a characteristic of MPH [5, 10, 26]. The central ONL and photoreceptor layer remain normally intact in MPH and TLH [5, 10]. The dehiscence of the central ONL (Fig. 3a) is likely caused by the large oblique anterior traction due to the elevation of the inner layers of the foveal walls. Figure 3c shows a TLH with LHEP in an eye with high myopia. The degeneration of the central ONL was likely caused by the large schisis between the OPL and ONL which produced a tractional disruption of Henle fibers followed by a degeneration of photoreceptor cells.

Figure 3d shows a foveal pseudocyst with a small LHEP which was generated by tangential traction exerted by ERM. The morphology of foveal pseudocysts is similar to that of TLH (elevation of the inner layers of the foveal walls, schistic splitting of the foveal walls between the OPL and HFL) with the exception that the horizontal layer of the Müller cell cone is not disrupted and keeps the elevated inner layers of the foveal walls together [5]. Figure 3e and f shows further cases of a foveal pseudocyst with LHEP. The pseudocyst in the eye with macular pucker shown in Fig. 3e was caused by traction exerted by ERM which produced a high thickening of the foveal walls. Anterior traction exerted by the thickened inner layers of the foveal walls onto the central outer retina produced the detachment from the RPE. The pseudocyst shown in Fig. 3f was likely formed by traction from the partially detached posterior hyaloid which adhered at the parafovea. The inner layers of the foveal walls were not elevated; therefore, the walls were not splitted between the OPL and HFL. The traction caused a distortion of the tissue which explains the increased reflectivity of the HFL in the right foveal wall and parafovea.

In the case shown in Fig. 3g, vitreomacular adhesions caused traction onto the fovea and parafovea; this was associated with the development of LHEP at the foveal walls and a loss of the integrity of the central photoreceptors, as indicated by the irregular reflectivities of the EZ and IZ lines. A hyperreflective membrane lied above the surface of the foveal pit; this membrane continued to the hyperreflective layers of the LHEP. A similar case of vitreomacular traction with LHEP at the foveal walls is shown in Fig. 3h. The traction caused an elevation of the foveal center which further developed into a FTMH associated with the formation of edematous cysts in the foveal walls; the FTMH also displayed LHEP at the vitreal surface of the foveal walls.

Examples of a mixed type of a MPH and DLH are shown in Fig. 3i and j. The straightening and thickening of the foveal walls are a characteristic of MPH [26], while the presence of LHEP and the degeneration of the central outer retina are a characteristic of DLH [5, 10]. The photoreceptor degeneration in the macula shown in Fig. 3j resulted from age-related macular degeneration. The ERM continued to the outer (Fig. 3i) or inner (Fig. 3j) hyperreflective layer of the LHEP. In the case of Fig. 3j, the LHEP enlarged within 64.5 months after the first visit and formed a plug which filled the central fovea.

A DLH with CME in one foveal wall is shown in Fig. 3k. In the case shown in Fig. 3l, a, CME developed from a fovea with LHEP, likely due to traction exerted by ERM and the posterior hyaloid which adhered at the ERM in the perifovea (*arrows* in Fig. 3l). Although the shape of the fovea recorded at the first visit appeared nearly normal, the traction produced a distortion of the right foveal wall and parafovea, as indicated by the increased reflectivity of the HFL. The CME was associated with a high elevation of the inner horizontal layer of the foveola and the inner layers of the foveal walls and parafovea (NFL to OPL). This produced a large schisis between the OPL and HFL, cystic cavities in the INL, and a tractional detachment of the central outer retina from the RPE. The photoreceptor outer segments were elongated in the detached area.

*Morphological alterations of DLH* It was described that DLH are morphologically stable and show only slow structural alterations over long time periods [8, 12, 17, 22–25]. However, relatively fast transient morphological alterations of foveas with DLH can be observed after which the foveal morphology spontaneously returns to the “regular” configuration of a DLH. The scan in Fig. 4a recorded at the first visit shows a disruption of the foveal structure due to CME, likely caused by tangential contraction of ERM. Edematous cysts in the foveola and within the INL of the foveal walls produced a large elevation of the inner layers of the foveal walls (NFL to IPL) and a detachment of the central photoreceptors from the RPE. The resolution of the cysts allowed a drop of the inner layers of the foveal walls and an almost normal restoration of the foveal morphology. Between 2 and 9.5 months, a DLH developed due to a degeneration of the INL and OPL in one foveal wall. Thereafter, a CME episode occurred again which was associated with the formation of LHEP at the vitreal surface of the foveal walls; the ERM continued to the inner hyperreflective layer of LHEP. After 29 months, the DLH was reestablished. The foveal center and the central surface of one foveal wall were covered by a tissue of medium reflectivity, likely composed of Müller cells (*arrow* in Fig. 4a). Between 37 and 42.5 months, a small FTMH developed due to the formation of edematous cysts in the INL and between the OPL and HFL [27] which caused a high elevation of the inner layers of the foveal walls (NFL to OPL) that produced an oblique anterior displacement and a detachment of the central outer retina from the RPE. Thereafter, the edematous cysts resolved and the FTMH closed spontaneously by a drop of the elevated inner layers of the foveal walls. At the end of the examination period, the fovea showed again a DLH configuration.

The DLH shown in Fig. 4b was characterized by a photoreceptor-free center of the foveola which was filled by a tissue of medium reflectivity, likely composed of Müller cells, a cavitation of the foveal pit into one elevated foveal wall, ERM and retinal folds at the surface of the opposite wall, and LHEP at the inner surface of the foveal walls and parafovea. Within 4.5 months after the first visit, the inner layers of the foveal walls elevated largely due to the formation of edematous cysts in the INL and between the OPL and HFL. There was a tissue of medium reflectivity at the central surface of one foveal wall which connected the Müller cells in the foveola with the LHEP at the edge of this wall (*arrows* in Fig. 4b). The resolution of the edematous cysts (5 months) allowed a drop of the elevated inner layers of the foveal walls and resulted in a regeneration of the foveal configuration to a morphology similar to that observed at the first visit. Thereafter, a reappearance of the edematous cysts elevated again the inner layers of the foveal walls. At the end of the examination period, the foveal configuration was similar to that observed at the initial visit.

The DLH shown in Fig. 4c contained photoreceptors throughout the foveal center at the first visit, as indicated by the nearly intact ELM and EZ lines. In the further course, the central photoreceptors degenerated, and the foveal center was composed of a tissue of medium reflectivity, likely Müller cells. These cells were connected by a tissue bridge to the LHEP at the inner surface of a foveal wall (*arrow* in Fig. 4c).

*DLH and FTMH* A DLH can be formed after closure of a FTMH and can evolve into a FTMH. A certain number of eyes with a FTMH showed the presence of LHEP at the vitreal surface of the foveal walls (Fig. 5a), suggesting that the FTMH developed from a DLH. Cases of a development of a DLH into a FTMH are shown in Fig. 5b–e. The development of a DLH is often caused by traction exerted by ERM (Fig. 5c, d) or vitreofoveal adhesion (Fig. 5e), while a FTMH likely develops when edematous cysts in the foveal walls are formed which cause a large elevation of the inner layers of the walls; this is associated with a dehiscence and detachment of the central outer retina from the RPE (Fig. 5c–e) [27, 28]. In the DLH shown in Fig. 5c (22 months), the foveal center was free of photoreceptors and composed of a tissue with medium reflectivity, likely Müller cells. In the case shown in Fig. 5d, vitrectomy with ILM and ERM peeling performed 31 months after the first visit resulted in a closure of the FTMH and a disappearance of the LHEP. However, the foveal center was largely devoid of photoreceptors and was filled by Müller cells. This tissue also covered the central surfaces of the foveal walls. Figure 5f shows an example of a development of a DLH after surgical closure of a FTMH. The FTMH closure was associated with a resolution of the edematous cysts which allowed a drop of the elevated foveal walls [29, 30].

*Foveal regeneration after surgical removal of LHEP* Fig. 6a–c shows cases of the foveal regeneration after surgical removal of LHEP by vitrectomy with ILM and ERM peeling. The DLH shown in Fig. 6a was characterized by large degenerative cavitations below the elevated inner layers of the foveal walls and a disruption of the central ONL, as indicated by the gap in the ONL and the ELM and EZ line defects. The surgical removal of the ERM and LHEP was associated with a drop of the inner layers of the foveal walls and a disappearance of the degenerative cavitations. The central ELM largely regenerated. However, there remained a gap in the central ONL and central defects of the EZ and IZ lines, suggesting that the centralmost fovea was free of photoreceptors. The foveal center was largely filled by a tissue of medium reflectivity, likely composed of Müller cells. This tissue covered also the central surface of the dorsonasal foveal wall.

The mixed type of a MPH and DLH shown in Fig. 6b displayed a widening of the central ONL and outward deflections of the ELM and EZ lines instead of inward deflections as in the normal fovea. The inner part of the foveola was filled by a tissue of medium reflectivity, likely Müller cells; this tissue continued to the LHEP at the rim of one foveal wall. The surgery caused a drop of the formerly elevated inner layers of the foveal walls, a disappearance of the degenerative cavitations, and a restoration of a nearly normal foveal pit. However, the foveal center was largely devoid of an ONL and photoreceptors and was formed by a tissue of medium reflectivity, likely Müller cells. This tissue also covered the central surfaces of the foveal walls. Similarly, the surgical closure of the FTMH shown in Fig. 6c, which likely developed from a DLH, produced a fovea with a gap in the central ONL that was filled by Müller cells.

## Discussion

The pathogenesis of DLH remains largely unclear. While TLH is generated by traction onto the fovea [13, 15, 19], DLH was suggested to be produced by a slow and chronic degenerative process which results in the formation of the cavitations into the lower foveal walls, the degeneration of the central photoreceptor layer and, in many cases, the development of LHEP at the vitreal surfaces of the foveal walls and parafovea [9]. In this study, we present various cases which show that tractional forces also play a primary pathogenic role in the development of DLH.

**Fig. 7:**
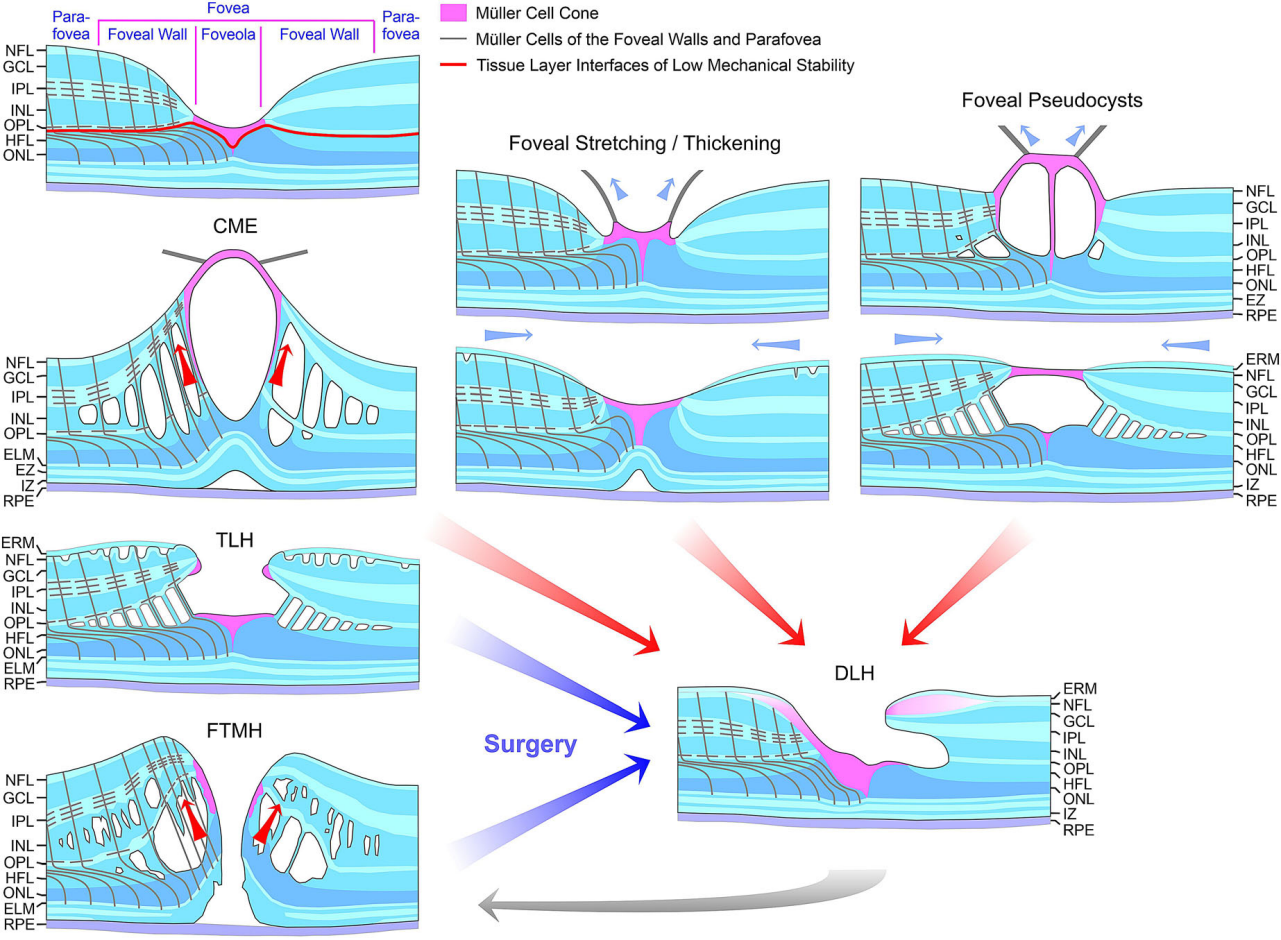
Schematic summary of different modes of the development of degenerative lamellar holes (DLH) induced by traction onto the fovea. The image *above* shows a schematic cross section through the normal fovea. The *blue arrows* indicate anterior hyaloidal traction and horizontal traction exerted by epiretinal membranes (ERM), respectively. The *red arrows* indicate anterior traction exerted by the stretched Müller cells of the foveal walls which causes a detachment of the central outer retina from the retinal pigment epithelium (RPE). The development of DLH may be preceded by cystoid macular edema (CME), which tractionally disrupts the foveal structure, or by traction onto the fovea exerted by the partially detached posterior hyaloid and/or ERM which causes a stretching and thickening of the foveola associated with or not a detachment of the central fovea from the RPE. The traction may also produce foveal pseudocysts. In addition, surgical treatment of tractional lamellar holes (TLH) or full-thickness macular holes (FTMH) may result in the development of DLH. A DLH may evolve into a FTMH. ELM, external limiting membrane; EZ, ellipsoid zone; GCL, ganglion cell layer; HFL, Henle fiber layer; INL, inner nuclear layer; IPL, inner plexiform layer; IZ, interdigitation zone; NFL, nerve fiber layer; ONL, outer nuclear layer; OPL, outer plexiform layer

We found that the formation of a DLH is preceded by a tractional deformation of the fovea due to the action of contractile ERM (Figs. 1b, g, h, 2c, and 4a) and (Figs. 1c, 2b)/or (Fig. 5e) the partially detached posterior hyaloid, or by traction exerted by edematous cysts (Fig. 2a). The tractional deformation of the fovea proceeded during months or years before the development of a DLH. A DLH may develop after an anterior stretching and thickening of the foveal center (Fig. 1h), from a foveal pseudocyst (Fig. 1b), after a tractional detachment of the foveola from the RPE (Fig. 1g), and after CME (Figs. 2a–c and 4a). Surgical treatment of a TLH (Fig. 1i) or a FTMH (Fig. 5f) may also result in the development of a DLH. A schematic summary of the different modes of DLH development found in this study is shown in Fig. 7.

It was suggested that the development of the degenerative cavitations in DLH is a two-step process: an initial tractional disruption of the Müller cell cone produces a schisis between the OPL and HFL of the foveal walls which is followed by a degenerative enlargement of the schisis to the cavitations of the foveal pit into the lower foveal walls [5]. This pathogenic model is reflected in the case shown in Fig. 1a. A tractional deformation of the fovea followed by the formation of a schisis between the OPL and HFL of the foveal walls is shown in Fig. 1d and e. The schisis may enlarge to degenerative cavitations by a degeneration of Henle fibers which are composed of photoreceptor axons surrounded by the outer processes of the Müller cells of the foveal walls [3, 5, 31]. The degeneration of Henle fibers was proposed to be followed by a degeneration of the photoreceptor synapses and a retrograde degeneration of horizontal and bipolar cells resulting in a degeneration of the OPL and INL [5]. The degeneration of Henle fibers also results in a degeneration of the photoreceptor cells in the central ONL which causes the photoreceptor layer defects in the foveola usually found in DLH [5]. The loss of the central photoreceptor cells may trigger the hypertrophy and proliferation of the cells of the disrupted Müller cell cone in the foveola which fill the spaces left by the degenerated photoreceptor cells and that contribute to the formation of LHEP [5]. However, a schisis between the OPL and HFL of the foveal walls may not be required for the subsequent development of degenerative cavitations, as indicated by the cases of vitreofoveal traction (Fig. 1c) and CME (Figs. 2b, c and 4a) which display a cystic disruption of the INL, but not between the OPL and HFL. On the other hand, in all cases of a tractional deformation of the fovea, a disruption of the Müller cell cone in the foveola seems to be a precondition of DLH formation.

DLH are considered to be clinically and morphologically stable and show only slow structural alterations over long time periods that include a slow increase in the degenerative cavitations and of the degeneration of the central outer retina (Fig. 1g, i–k) [5, 8, 12, 17, 22–25]. Here, we describe cases in which the foveal configuration of a DLH was spontaneously reestablished after fast transient episodes of CME (Figs. 2a and 4a, b) and the formation of a small FTMH (Fig. 4a). The relative stability of a DLH, which readily reestablishes after fast morphological alterations of the fovea, may support the assumption that DLH are a kind of healing response to stabilize the foveal morphology in cases of a tractional disruption of the foveal integrity.

The pathogenesis and functional role of LHEP are unknown. In the present study, we show that different types of foveal defects may exhibit LHEP, including CME (Figs. 3k, l and 4a), vitreomacular traction syndrome (Fig. 3g, l), foveal pseudocysts (Fig. 3d–f), TLH (Fig. 3c), and FTMH (Figs. 3h, 4a, 5a–d and 6c). We also show cases of a MPH with cleaved edges (Fig. 3a, b) and mixed types of MPH and DLH with LHEP (Figs. 3i, j and 6b). Thus, LHEP is not restricted to DLH, although the highest incidence is found in DLH.

It was suggested that the formation of LHEP is a secondary event following the development of a lamellar hole and may represent an attempt to protect the fovea from the traction exerted by contractile ERM and/or the partially detached posterior hyaloid which induces the development of the holes [32]. In some cases, the retinal folds produced by contractile ERM became smaller after the development of LHEP (Fig. 1g), while this was not observed in other cases (Fig. 4a). A decrease in retinal folds may support the assumption that LHEP are formed to counteract the tractional forces. On the other hand, most cases of foveal pseudocysts, TLH, and MPH have no LHEP although these foveal defects are tractionally formed [5]. Therefore, we assume that the formation of LHEP cannot be explained only by the traction onto the fovea.

Tractional ERM and hyaloidal membranes contribute to the development of LHEP [5, 13, 15, 17, 18, 21]. We show various cases in which ERM were present before the development of LHEP (Fig. 1b, g, h, i, j). The hyperreflective ERM may continue to the inner (Fig. 1c), outer (Fig. 3a, b) or both hyperreflective layers of LHEP (Fig. 1b). The presence of connections between the Müller cells in the foveola and LHEP found in many cases of DLH (Figs. 1c, f–h, 2a, c, and 4a–c) may support the assumption that, in addition to vitreal cells like fibroblasts and hyalocytes, hypertrophied and/or proliferating cells of the disrupted Müller cell cone contribute to the development of LHEP [5, 15, 17, 18, 21]. Because such connections between Müller cells in the foveola and LHEP are present preferentially in nonelevated foveal walls without degenerative cavitations (Figs. 1f, i, k, 2c, and 4b), it was suggested that they are formed to prevent an elevation of the inner layers of the foveal walls which otherwise may contribute to the enlargement of the cavitations [5]. We suggest that, in addition to the tractional deformation of the fovea, the disruption of the Henle fibers and the degeneration of the central photoreceptor cells are events which trigger the hypertrophy and proliferation of the cells of the disrupted Müller cell cone in the foveola and the formation of LHEP in DLH. Foveal pseudocysts, TLH, and MPH do normally not display a degeneration of Henle fibers [5]; this may explain the low incidence of LHEP in these types of foveal defects. It is suggested that the development of a DLH is a retinal wound repair process after a tractional disruption of the Müller cell cone and a degeneration of Henle fibers, to prevent a further increase in the degenerative cavitations. Further research is required to reveal the etiologies and pathogenic steps implicated in the development of DLH.

It was found that the presence of LHEP in eyes with lamellar holes is associated with greater photoreceptor layer defects and poor visual acuity [8, 13, 33, 34]. Eyes with lamellar holes and LHEP have also a poorer visual outcome after vitrectomy than eyes with lamellar holes and no LHEP, because of a greater proportion of a failure of a regeneration of the photoreceptor layer [33]. Both regular and irregular anatomical outcomes of the surgical treatment of DLH were described [16, 33, 35–37]. In the two cases of a surgical treatment of a DLH described in this study (Fig. 6a, b), the degenerative cavitations into the lower foveal walls disappeared after surgery which was associated with a drop of the previously elevated inner layers of the foveal walls. However, in the cases of a surgical removal of LHEP (Figs. 5c and 6a–c), surgery was followed by an irregular regeneration of the fovea, i.e., the foveal center was devoid of photoreceptors and was filled by a tissue of medium reflectivity, likely composed of hypertrophied and/or proliferating cells of the disrupted Müller cell cone. The absence of photoreceptors in the foveal center may explain the poor visual outcome of eyes with lamellar holes and LHEP after vitrectomy. A similar irregular regeneration of the fovea was described in several cases of a surgical closure of FTMH [30]. The reason for the irregular foveal regeneration is unclear. The degeneration of Henle fibers during the development of the degenerative cavitations in DLH is associated with a degeneration of central photoreceptor cells [5] which may explain in part the lack of photoreceptors in the foveal center after surgery.

A regular regeneration of the fovea after spontaneous or surgical closure of FTMH was shown to involve in many cases a centripetal displacement of the central ONL, likely mediated by horizontal traction exerted by the Müller cells of the foveal walls; this displacement closes the gap in the ONL and increases the central photoreceptor density [29, 30]. Such a centripetal displacement of photoreceptor cells was not observed in the cases described in this study. It could be that the time period after surgery was too short to observe such a displacement because it was shown that the displacement may proceed after a considerable time delay of many months after hole closure [30]. Another explanation may be that the tissue of medium reflectivity, which fills the ONL-free part of the foveal center and that is likely composed of cells of the disrupted Müller cell cone, inhibits the centripetal displacement of the ONL. Further investigations are required to determine the mechanisms of the regular and irregular regeneration of the fovea after hole closure.

A DLH can evolve to a FTMH (Fig. 5b–e); this is associated with the formation of edematous cysts in the foveal walls which further elevate the inner layers of the walls [5, 27, 38, 39]. Development of edematous cysts occurs, for example, under inflammatory conditions, e.g., after cataract surgery [40, 41]. Previous studies reported a high incidence of FTMH formation after surgical peeling of LHEP [15, 17]. Because LHEP is connected to the Müller cells of the foveola (Figs. 1c, f–h, and 4a–c) [5], peeling of LHEP may also remove the central Müller cells which seal the defects of the central outer retina, thus increasing the likelihood of a disruption of the foveal center and FTMH formation.

In summary, we show that DLH develop after tractional deformations of the fovea for months or years due to the action of ERM and/or the posterior hyaloid, or by CME. DLH may be also formed by surgical treatment of TLH or FTMH. A DLH can be spontaneously reestablished after short transient episodes of CME and small FTMH and can evolve to a FTMH. Surgical treatment of a DLH may result in an irregular regeneration of the foveal center without photoreceptors.

## Abbreviations

BCVA: Best-corrected visual acuity
CME: Cystoid macular edema
DLH: Degenerative lamellar hole
ELM: External limiting membrane
ERM: Epiretinal membrane
EZ: Ellipsoid zone
FTMH: Full-thickness macular hole
GCL: Ganglion cell layer
HFL: Henle fiber layer
ILM: Internal limiting membrane
INL: Inner nuclear layer
IPL: Inner plexiform layer
IZ: Interdigitation zone
LHEP: Lamellar hole-associated epiretinal proliferation
MPH: Macular pseudohole
NFL: Nerve fiber layer
ONL: Outer nuclear layer
OPL: Outer plexiform layer
RPE: Retinal pigment epithelium
SD-OCT: Spectral-domain optical coherence tomography
TLH: Tractional lamellar hole
